# Characterizing the mechanics of rectangular peg–hole disassembly and the effect of the active compliance centre on the extraction force

**DOI:** 10.1098/rsos.240956

**Published:** 2024-11-27

**Authors:** Farzaneh Goli, Ali Aflakian, Mo Qu, Yue Zang, Mozafar Saadat, Duc Truong Pham, Yongjing Wang

**Affiliations:** ^1^ Mechanical Engineering Department, University of Birmingham, Birmingham, UK

**Keywords:** quasistatic, robotic disassembly, remanufacturing, active compliance, rectangular peg–hole disassembly

## Abstract

This paper aimed at facilitating robotized disassembly for remanufacturing by focusing on the challenge of rectangular peg–hole disassembly. The study explores all potential contact states during the rectangular peg–hole disassembly process and identifies 26 distinct conditions, 16 of which are related to jamming. The contact conditions are categorized into five groups based on the number of contacts with the surface. Thereafter, it provides an in-depth analysis of jamming phenomena during the extraction process, employing both geometrical and quasistatic analyses to establish boundary conditions for jamming. Furthermore, the efficacy of the active compliance centre position in preventing jamming area is explored, considering critical variables such as compliance degree, centre location and initial position errors. The outcomes highlight that positioning the compliance centre at the end of the peg is the most effective strategy for reducing the jamming area and extraction force. Finally, the simulated results are confirmed by experiments and demonstrated 77.1% reduction to the maximum extraction force with the correct active compliance centre position, as opposed to when it is placed at the top of the peg. The findings contribute insights into the intricate dynamics of disassembly, revealing potential avenues for optimizing automated robotic systems in remanufacturing.

## 1. Introduction

The disposal of end-of-life (EOL) products creates both environmental and economic challenges [1]. To address these challenges, EOL products can undergo processes of remanufacturing, ensuring non-destructive separation of components and materials for prospective reuses [2].

Remanufacturing, a multi-stage process involving disassembly, cleaning, inspection, repair or replacement of damaged parts, reassembly and testing, has emerged as a pivotal link connecting product return with product recovery [3,4]. However, the manual disassembly process has proven to be inefficient, prompting the adoption of automatic disassembly facilitated by robots to enhance efficiency, reduce time and labour costs, and mitigate the dangers associated with human involvement [5]. A particular challenge in disassembly is the extraction of pegs from clearance-fit holes [6].

This routine operation, prevalent in the disassembly of mechanical components such as shaft–hub connections in machinery, gears and sprockets, and pump and motor couplings, extends to the dismantling of assemblies with rectangular pegs and holes. While previous research has extensively explored the peg–hole assembly, disassembly presents unique challenges due to the reverse nature of the process. Unlike assembly of new products, which is deterministic because the components to be assembled are of known geometries, dimensions and states, disassembly is stochastic as it has to contend with used products of uncertain shapes, sizes and conditions. They introduce uncertainties in tolerances and misalignments between the peg and hole in separation processes and may lead to increased extraction forces and potential damage to the components [7]. Lateral and angular misalignment between separated components can amplify reaction forces, resulting in problems during disassembly, including jamming and wedging [8].

It should be mentioned that in robotic disassembly, jamming describes the peg becoming lodged at an angle, complicating disassembly with misalignment issues, while wedging refers to the peg becoming tightly stuck in the hole due to friction or deformation. The use of robots in peg–hole disassembly should consider jamming and wedging due to unbalanced forces, which can cause damage to components and robots [8].

To address these challenges, researchers have explored various strategies, such as the remote compliance centre (RCC) [9–12], to avoid jamming and wedging during disassembly [10,11]. Despite these efforts, there is a notable absence of comprehensive investigations into multiple peg–hole disassembly, leaving a gap in understanding the mechanisms and contacts involved in this process. Simunovic [13] and Nevins & Whitney [14], in their exploration of peg–hole assembly dynamics, employed a compliant manipulator, advancing the field by introducing an RCC device to enhance precision and efficiency. The investigation extended to various dimensions, encompassing rectangular peg insertion without chamfers [15] and three-dimensional scenarios [16,17]. Sturges *et al*. contributed to the discourse by developing the spatial remote compliance centre (SRCC) [18]. Strip [19] innovatively devised a hybrid force–position strategy tailored for three-dimensional convex pegs, integrating active compliance. Zhang *et al*. investigated the intricacies of peg–hole disassembly by conducting a quasistatic analysis with a compliance device. Their inquiry scrutinized critical variables such as compliance degree, the position of the compliance centre and positional errors [11]. Wang *et al*. undertook a comparative study of pitting passive against active compliance and concluded that the latter, despite its costs and restricted response speed, outshines in improving the dynamic response and assembly reliability [20].

This paper builds on prior studies [11,21] that investigated contact states within one-peg and one-hole scenarios.

These scenarios are characterized by limited contact points concentrated at the peg tip and the hole’s inner surface. In [7,22], we also explored the extension of techniques from single peg–hole to dual peg–hole assemblies, indicating the potential for more accurate robotic systems. This study underscores the potential applicability of single peg–hole techniques, such as the compliance centre approach, to the dual peg–hole case, with experimental results supporting the proposed claim.

The complexities of rectangular peg–hole scenarios are less explored in the literature. These kinds of pegs and their mating holes are commonly utilized in mechanical products, including industrial components, furniture, toys and electronic devices. One of the complexities and differences in such configurations, compared with cylinder peg–hole assemblies, arises from the fact that a rectangular peg can establish multiple contact states with its corresponding hole, leading to complex jamming conditions.

While cylindrical pegs typically exhibit rotational symmetry, reducing the variety of contact points during extraction, rectangular pegs can contact the hole on multiple planes and edges. This significantly increases the complexity of disassembly for rectangular pegs, making control of lateral and angular misalignment more critical. Cylindrical peg–hole configurations generally require less extraction force and experience minimal friction. The geometry and symmetry of the cylinder make alignment, control and sensing easier, often demanding precise tolerances but with predictable forces. By contrast, disassembly tasks involving polygonal shapes face more complex forces, higher friction and require more advanced robotic control strategies due to the increased complexity of surface interactions and mechanical constraints.

The extraction force required to remove a rectangular peg from a mating hole constitutes another critical complexity within such configurations. Dismantling these assemblies typically demands substantial force, rendering the process challenging, time-consuming and costly. A deep understanding of the mechanics involved in rectangular peg–hole disassembly becomes essential for enhancing the efficiency of disassembly processes. In this study, the mechanics of rectangular peg–hole disassembly are investigated, and the effect of the active compliance centre (ACC) position on the extraction force in three dimensions is analysed. Specifically, the relationships between the geometrical parameters of the peg and hole, the frictional properties of the contact surfaces, and the extraction force required to remove the peg from the hole are characterized. Thereafter, the effect of the ACC position on the extraction force is explored, and the optimal ACC position that minimizes the force required for disassembly is identified. The geometrical boundary conditions (specifically, depths of extraction) of jamming are highlighted, and a solution using ACC to be minimizing the jamming area is proposed.

**Figure 1.**
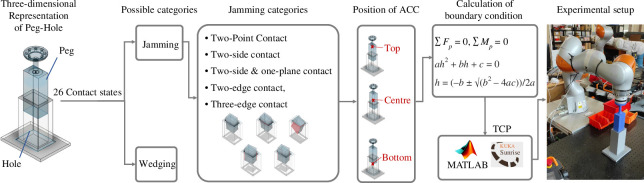
Investigating the impact of the active compliance centre (ACC) position on 16 jamming conditions among 26 possible contact situations both in simulations and experiments.

Figure 1 illustrates the structure of this study, which delves into the robotic removal of a three-dimensional rectangular peg from a hole. Two categories of contact states are listed, jamming and wedging, which are situations where the peg cannot be extracted from the hole smoothly. This study explores the influence of ACC positions on 16 jamming conditions using a formula derived from quasistatic analysis. Finally, the Kuka LBR robot is utilized to validate the theoretical approach for practical application.

Our findings provide insights into the mechanics of rectangular peg–hole disassembly and highlight the importance of considering the ACC position in optimizing disassembly processes. This knowledge can be applied to a wide range of mechanical systems and can lead to significant improvements in efficiency, cost and sustainability.

Section 2 provides an analysis of rectangular peg extraction during disassembly, encompassing geometric analysis and delves into the force analysis of rectangular peg–hole extraction as a methodology. Section 3 presents the results and discussion of the analysis. Section 4 details the experimental design and results, confirming the theoretical disassembly model.

## 2. Methodology

Extracting a rectangular peg from a hole is a common task in disassembly applications. Considering the forces and moments involved in the extraction of a rectangular peg from a hole is necessary to successfully complete the common task of extracting the peg from the hole. This analysis is based on the following main assumptions:

— The pegs are stiff.
— There is only one extraction direction: vertical upwards.
— The ACC is located along the axis of extraction and the hole.
— The stiffness parameters ($K_{X},K_{Y}$ and $K_{\theta },K_{\Phi },K_{\psi }$) define the resistance of the system to lateral and rotational displacements during extraction. Higher stiffness values correspond to lower compliance, leading to reduced rotations and minimized two-contact regions.
— Adhesive forces, such as parts being bonded by glue or due to corrosions, are not considered.

Predicting force and torque during disassembly helps us identify configurations in which compliant devices supporting the pegs, such as RCC, SRCC or ACC, may fail to avoid wedging and jamming [23]. This information provides a better understanding of the characteristics of rectangular peg–hole disassembly using compliance mechanisms.

The forces and moments acting on the peg can be used to predict the likelihood of wedging and jamming during extraction. If not properly controlled, the peg can wedge in the hole or jam against the sides of the hole. This knowledge can be used to design and control extraction processes to minimize the risk of these problems.

In a rectangular peg–hole problem, the use of an ACC is expected to result in successful extraction with an appropriate initial configuration. The ACC will mitigate the likelihood of wedging and jamming by adjusting the forces and moments acting on the peg in real time. This allows for a smooth and safe extraction process. The extracting phase of the rectangular peg–hole extraction problem is thoroughly examined in the following sections.

### 2.1. Geometric analysis

**Figure 2.**
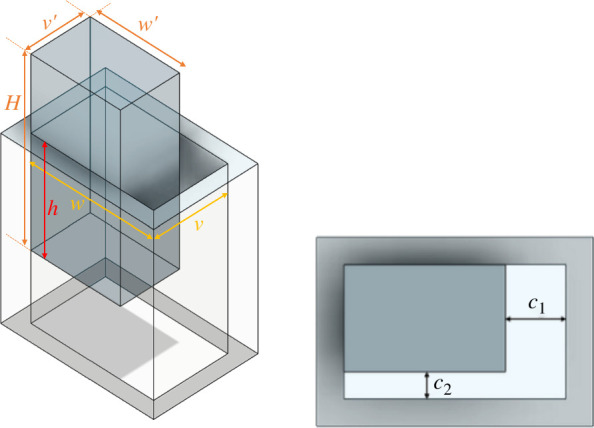
Geometric model of a rectangular peg–hole.


The main dimensions of a rectangular peg–hole are shown in figure 2. The hole length and width are w and v, respectively, and the peg length and width are $w^{{,}^{\prime }}$ and $v^{{,}^{\prime }}$, respectively. The distance between the peg and the hole in the rectangle’s length is ${∁}_{1}$, the distance between the peg and the hole in its width is ${∁}_{2}$, and h represents the current extraction depth:

(2.1) $${∁}_{1}=w{-w}^{\prime }\;\text{and}\;{∁}_{2}=\;v-v^{\prime }.$$

Due to incompatible dimensions, the peg cannot be extracted from the hole in some cases. The rectangular peg can be extracted from the hole if the following condition is met:


(2.2) $$\begin{array}{r} w^{\prime }<w\;\text{and}\;v^{\prime }<v. \end{array}$$

The ability of a peg to be extracted from its hole is referred to as extractability.

**Figure 3.**
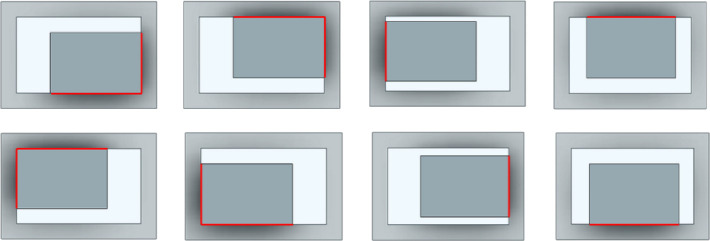
Maximum left/right- and down/upside travel cases.

Figure 3 illustrates the rectangular peg–hole that is removable based on its maximum left or right or left and right displacements. The pegs undergo three types of rotation—yaw, pitch and roll—while maintaining their current contact positions to generate various potential contact states. Disassembly of the rectangular peg from the corresponding hole can lead to a total of 26 potential contact states; however, after eliminating redundant states (with the same initial conditions), 16 distinct contact states remain (table 1). These contact states can be categorized into five primary groups: (i) no contact, (ii) point contact, (iii) line contact, (iv) combined point and line contact and (v) combined line and plane contact.

**Table 1.** Various contact states for rectangular peg–hole extractions.

	one-contact state	two-contact state	three-contact state
point contact	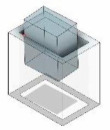	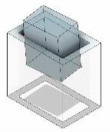	
line contact	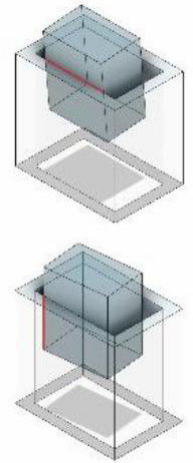	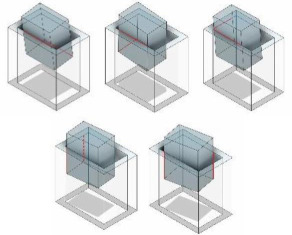	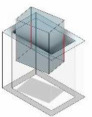
point and line contact		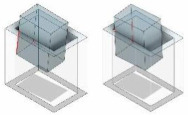	
line and plane contact		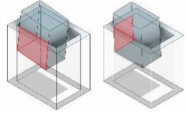	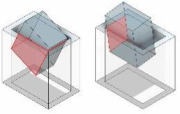

Ideally, the desired state for the peg is one of the states in the ‘no contact’ group; nevertheless, in practice, this state is rarely achieved due to the small clearance between the peg and hole. The disassembly process typically commences in a two-contact state, attributed to the compliant nature of manipulation and the presence of lateral and angular errors. Such a two-contact state occurs when a compliant manipulator grasps the peg, leading to slight shifts and rotations. As the peg is progressively extracted, compliance may help reduce the errors, and the process can transition into one-contact states or states involving line or plane contact.

**Figure 4.**
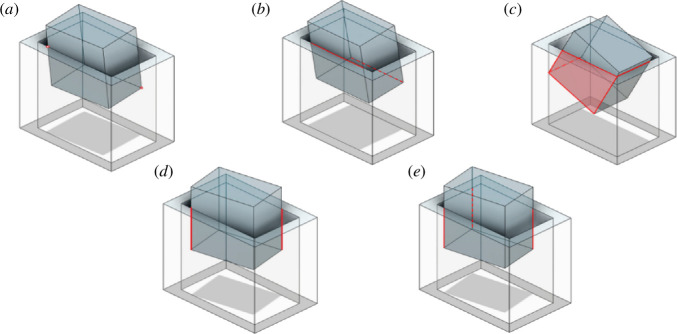
Typical states of the rectangular peg–hole disassembly process: (*a*) two-point contact, (*b*) two-sided contact, (*c*) combined two-sided and one-plane contact, (*d*) two-edge contact and (*e*) three-edge contact.

In rectangular peg–hole disassembly, jamming is observed within 8 out of the 16 distinct contact modes. These states can be categorized into five primary groups: (i) two-point contact, (ii) two-sided contact, (iii) combined two-sided and one-plane contact, (iv) two-edge contact, and (v) three-edge contact, as shown in figure 4.

Jamming is a prevalent challenge in the context of peg–hole disassembly. Jamming arises when a peg becomes immobile due to forces and moments that are incorrectly applied. This issue primarily manifests in the two-contact state and can be mitigated by reducing the extent of the two-contact area and by carefully controlling the location at which the two-contact state occurs [24]. Notably, the concern of jamming is relatively minimal in cases of four-line contact, as it manifests as wedging rather than jamming occurring at specific angles. Sturges studied four-point contacts during peg–hole insertion, also known as wedging [16,17]. This study is centred on the analysis of jamming within two- and three-contact states, recognizing the distinct nature of the issue in these scenarios.

### 2.2. Force analysis of rectangular peg extraction

Analysing the forces and moments involved in the extraction of a rectangular peg is crucial for planning precise motions. The insertion of peg into hole followed the same geometric analysis as extraction. Successful removal of the rectangular peg from its corresponding hole hinges on meeting specific conditions. Throughout the extraction process, the rectangular peg encounters various contact states, some of which are more favourable than others.

As explained previously, in the context of a three-dimensional rectangular peg–hole problem, a total of 16 potential states can be identified (table 1). Among these states, eight are linked to contact situations that may lead to jamming (figure 4), and these eight states have been categorized into five distinct groups, as elucidated in §2.1. This subsection will proceed to conduct an in-depth analysis of each of these categorized groups.

**Figure 5.**
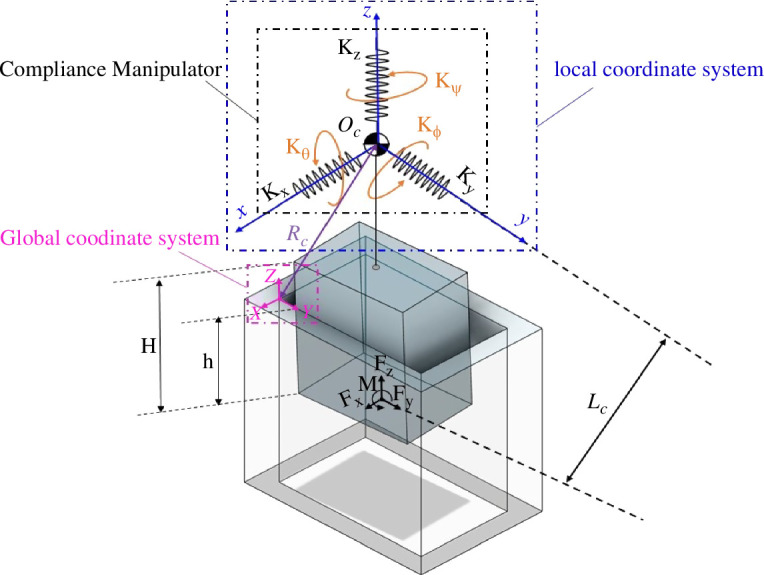
Dimensions and reference frame for the rectangular peg–hole.

Considering the rectangular peg–hole system as modelled in figure 5, a consistent reference frame is utilized for the rectangular peg throughout all states, positioned at the end of the peg. All the forces and moments applied to the pegs are specified with respect to this established reference frame. Now, consider rotation angles θ around the *X*-axis, ϕ around the *Y*-axis and ψ around the *Z*-axis.

It is assumed that both the peg and the hole are initially in a jamming state, and for the sake of computational simplification, it is further assumed that angular deviations are relatively small. This assumption holds valid in practice, as the peg’s angular errors typically remain within a few degrees at most.

**Figure 6.**
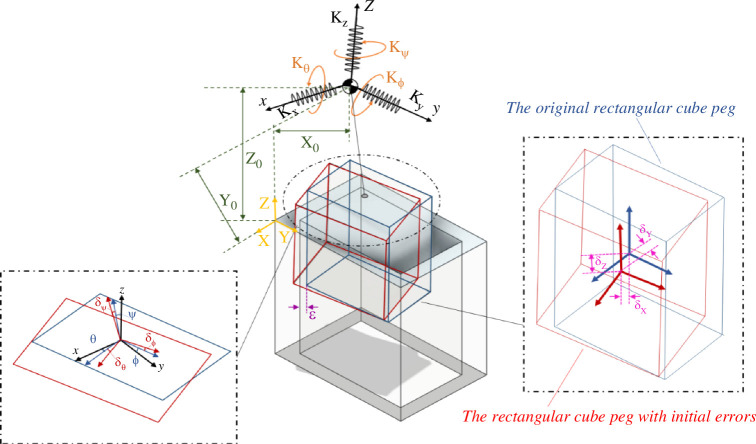
Definition of initial errors and position in the rectangular peg–hole.

As depicted in figure 6, $\vec{\delta }=[\delta_{X},\delta_{Y},\delta_{Z},\delta_{\theta },\delta_{\phi },\delta_{\psi }{]}^{\prime }$ corresponds to the initial errors, and $\vec{r_{0}}=[X_{0},\;Y_{0},\;Z_{0},\;\theta_{0},\;\phi_{0},\;{\;\psi }_{0}{]}^{\prime }$ denotes the initial distances from the axis of the hole to the compliance centre and the peg tip.

The initial state of the compliance centre moves concurrently with the manipulator, and the new position of the compliance centre can change the configuration of the springs. The position and orientation used are considered relative to the coordinate reference system that is related to the hole. The coordinates of the compliance centre are $\vec{r_{m}}=[X_{m},\;Y_{m},\;Z_{m},\;\theta_{m},\;\phi_{m},\;{\;\psi }_{m}{]}^{\prime }$.

The disparity between the new state of the compliance centre and its initial state gives rise to state errors, defined as


(2.3) $$\begin{array}{r} \vec{\delta }=\vec{r_{0}}-\vec{r_{m}}. \end{array}$$

It can be asserted that the linear and torsional springs undergo greater changes when the compliance centre’s initial position is more distant from the hole axis, and the rotation angle is larger after extracting the peg from the hole.

In addition, due to the differences in states between the peg’s state and the manipulator, the manipulator creates forces and torques, which are defined as follows:


(2.4) $$\begin{array}{r} \vec{f}=K\vec{\Delta }. \end{array}$$

In which $\vec{f}={\left[f_{sX},f_{sY},f_{sZ},\tau_{s\theta },\tau_{s\phi }{,\tau }_{s\psi }\right]}^{\prime }$ is the force/torque vector, *K* = diag[$k_{X},k_{Y},k_{Z},k_{\theta },k_{\phi }$, $k_{\psi }$] is the gain matrix and $\vec{\Delta }\;=\vec{\delta }+\vec{r}-\vec{r_{0}}$ , in which *r* is the current distance from the axis of the hole to the compliance centre and the peg tip.

#### 2.2.1. Case 1: two-point contact

In this case, we scrutinize the contact scenario where two points of the peg contact the surfaces of the hole (figure 4*a*).

In this case, the coordinate transformation matrix is formulated as follows:


(2.5) $$T_{p}^{h}=\left[\begin{array}{ccc} c\psi c\phi & s\theta c\psi s\phi -s\psi c\theta & s\theta s\psi +c\theta c\psi s\phi \\ s\psi c\phi & s\theta s\psi s\phi +c\psi c\theta & c\theta s\psi s\phi -s\theta c\psi \\ -s\phi & c\psi s\theta & c\phi c\theta \end{array}\right],\;T_{h}^{p}=\left[\begin{array}{ccc} c\psi c\phi & s\psi c\phi & -s\phi \\ s\theta c\psi s\phi -s\psi c\theta & s\theta s\psi s\phi +c\psi c\theta & c\psi s\theta \\ s\theta s\psi +c\theta c\psi s\phi & c\theta s\psi s\phi -s\theta c\psi & c\phi c\theta \end{array}\right].$$

A kinematic constraint in the closed-loop kinematic chain is characterized based on the spatial arrangement of the peg relative to the hole:


(2.6) $$R_{1}-T_{p}^{h}r_{1}=R_{C},R_{2}-T_{p}^{h}r_{2}=R_{C},$$

where


(2.7) $$R_{1}=\left[\begin{array}{c} 0\\ \varepsilon_{1}\\ 0 \end{array}\right],\;R_{2}=\left[\begin{array}{c} -v\\ w-\varepsilon_{2}\\ -h^{\prime } \end{array}\right],\;r_{1}={\left[\begin{array}{c} \frac{v^{\prime }}{2}\\ -\frac{w^{\prime }}{2}\\ -\left(H+L_{C}-h\right) \end{array}\right]}_{p},\;r_{2}={\left[\begin{array}{c} -\frac{v^{\prime }}{2}\\ \frac{w^{\prime }}{2}\\ -\left(H+L_{C}\right) \end{array}\right]}_{p}.$$

**Figure 7.**
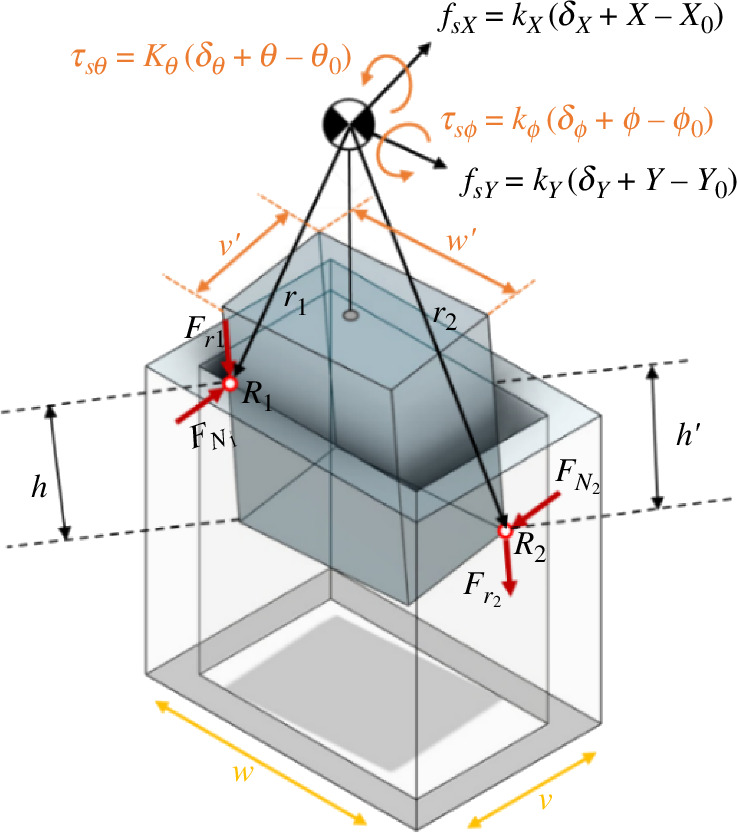
Geometry and forces during two-point contact in the rectangular peg–hole.

Therefore,


(2.8) $$\begin{array}{r} T_{p}^{h}\;\left(r_{1}-r_{2}\right)+\;R_{2}-\;R_{1}=0, \end{array}$$

where $\varepsilon_{1}$ represents the distance along the *Y*-axis from the left wall and $\varepsilon_{2}$ denotes the distance from the right wall. Additionally, $h^{{,}^{\prime }}$ signifies the vertical distance from the second contact point to the upper surface of the hole (figure 7).

Conducting a thorough force analysis involves considering the forces and torques from the peg–hole contact, as well as those at the compliance centre due to spring length variations. This analysis allows for the determination of the force $F_{z}$ in relation to other variables.

The frictional force and the applied force are as follows:


(2.9) $$F_{N1}=-F_{N1}T_{h}^{p}{\hat{e}}_{h}\times {\hat{e}}_{p}=F_{N1}{\left[\begin{array}{c} -s\theta s\psi s\phi -c\psi c\theta \\ s\psi c\phi \\ 0 \end{array}\right]}_{p}\;,\;F_{N2}=F_{N2}T_{h}^{p}\hat{I}=F_{N2}{\left[\begin{array}{c} c\psi c\phi \\ s\theta c\psi s\phi -s\psi c\theta \\ s\theta s\psi +c\theta c\psi s\phi \end{array}\right]}_{p}$$

(2.10) $$F_{r1}=-\mu F_{N1}\hat{k},\;F_{r2}=-\mu F_{N2}\left[\begin{array}{c} -s\phi \\ c\psi s\theta \\ c\phi c\theta \end{array}\right]=-\mu F_{N2}\hat{K}.$$

Considering $\sum F_{p}=0$ and $\sum M_{p}=0$, the following is the applied force and moment at the compliance centre:


(2.11) $$F+T_{h}^{p}f_{s}+F_{N1}+F_{N2}+F_{r1}+F_{r2}=0,$$

(2.12) $$M+r_{1}\times \left(F_{N1}+F_{r1}\right)+r_{2}\times \left(F_{N2}+F_{r2}\right)+T_{h}^{p}\tau_{s}=0,$$

in which $F={\left[F_{x},F_{y},F_{z}\right]}^{\prime },\;f_{s}=[f_{sx},f_{sy},f_{sz}],\;M={\left[M_{x},M_{y},M_{z}\right]}^{\prime },\;\tau_{s}=[\tau_{s\theta },\;\tau_{s\phi },\;\tau_{s\psi }\;]$.

In instances involving small clearances between the peg and hole, equations (2.11) and (2.12) can be formulated as


(2.13) $$\left[\begin{array}{c} F_{x}\\ F_{y}\\ F_{z} \end{array}\right]+\left[\begin{array}{ccc} 1 & \psi & -\phi \\ -\psi & 1 & \theta \\ \phi & -\theta & c\phi c\theta \end{array}\right]\left[\begin{array}{c} f_{sX}\\ f_{sY}\\ f_{sZ} \end{array}\right]+F_{N1}\left[\begin{array}{c} -1\\ \psi \\ 0 \end{array}\right]+F_{N2}\left[\begin{array}{c} 1\\ -\psi \\ \phi \end{array}\right]+\left[\begin{array}{c} 0\\ 0\\ -\mu F_{N1} \end{array}\right]-\mu F_{N2}\left[\begin{array}{c} -\phi \\ \theta \\ 1 \end{array}\right]=0,$$

(2.14) $$\left[\begin{array}{c} M_{x}\\ M_{y}\\ M_{z} \end{array}\right]+r_{1}\times \left[\begin{array}{c} -F_{N1}\\ \psi F_{N1}\\ -\mu F_{N1} \end{array}\right]+r_{2}\times \left[\begin{array}{c} F_{N2}+\phi \mu F_{N2}\\ -\psi F_{N2}-\theta \mu F_{N2}\\ \phi F_{N2}-\mu F_{N2} \end{array}\right]+\left[\begin{array}{ccc} 1 & \psi & -\phi \\ -\psi & 1 & \theta \\ \phi & -\theta & c\phi c\theta \end{array}\right]\left[\begin{array}{c} \tau_{s\theta }\\ \tau_{s\phi }\\ \tau_{s\psi } \end{array}\right]=0.$$

To solve the equations, the force $F_{z}$ is applied in the *z* direction ($F_{X},\;\;F_{Y}=0$), and compliance is incorporated along the *y* and θ directions ($f_{sZ}=0$):


(2.15) $$F_{z}=-\phi f_{sX}+\theta f_{sY}-\psi F_{N1}+\psi F_{N2}+\theta \mu F_{N2},$$

(2.16) $$\left[\begin{array}{c} F_{N1}\\ F_{N2} \end{array}\right]={\left[\begin{array}{cc} -1 & 1+\phi \mu \\ \psi & -\psi -\theta \mu \end{array}\right]}^{-1}\left[\begin{array}{c} -f_{sX}-\psi f_{sY}\\ \psi f_{sX}-f_{sY} \end{array}\right]=\frac{\left[\begin{array}{cc} -\psi -\theta \mu & -1-\phi \mu \\ -\psi & -1 \end{array}\right]}{\theta \mu -\psi \phi \mu }\left[\begin{array}{c} -f_{sX}-\psi f_{sY}\\ \psi f_{sX}-f_{sY} \end{array}\right].$$

By using equations (2.15) and (2.16), we have


(2.17) $$\begin{array}{c} \begin{array}{r} \left[\begin{array}{c} M_{x}\\ M_{y}\\ M_{z} \end{array}\right]=-\left[\begin{array}{c} \frac{w^{\prime }}{2}\mu F_{N1}+\left(H+L_{C}-h\right)\psi F_{N1}\\ \left(H+L_{C}-h\right)F_{N1}+\frac{v^{\prime }}{2}\mu F_{N1}\\ \frac{v^{\prime }}{2}\psi F_{N1}-\frac{w^{\prime }}{2}F_{N1} \end{array}\right]\\ -\left[\begin{array}{c} \frac{w^{\prime }}{2}\left(\phi F_{N2}-\mu F_{N2}\right)-\left(H+L_{C}\right)\left(\psi F_{N2}+\theta \mu F_{N2}\right)\\ -\left(H+L_{C}\right)\left(F_{N2}+\phi \mu F_{N2}\right)+\frac{v^{\prime }}{2}\left(\phi F_{N2}-\mu F_{N2}\right)\\ \frac{v^{\prime }}{2}\left(\psi F_{N2}+\theta \mu F_{N2}\right)-\frac{w^{\prime }}{2}\left(F_{N2}+\phi \mu F_{N2}\right) \end{array}\right]-\left[\begin{array}{ccc} 1 & \psi & -\phi \\ -\psi & 1 & \theta \\ \phi & -\theta & 1 \end{array}\right]\left[\begin{array}{c} \tau_{s\theta }\\ \tau_{s\phi }\\ \tau_{s\psi } \end{array}\right]. \end{array} \end{array}$$

In this scenario, when $F_{N2}=0$, it signifies the limit state of contact. Consequently, the boundary conditions can be established as follows:


(2.18) $$\left[\begin{array}{c} 0\\ 0\\ F_{z} \end{array}\right]+\left[\begin{array}{ccc} 1 & \psi & -\phi \\ -\psi & 1 & \theta \\ \phi & -\theta & 1 \end{array}\right]\left[\begin{array}{c} f_{sX}\\ f_{sY}\\ f_{sZ} \end{array}\right]+\left[\begin{array}{c} -F_{N1}\\ \psi F_{N1}\\ -\mu F_{N1} \end{array}\right]=0,$$

(2.19) $$f_{sX}+\psi f_{sY}-\phi f_{sZ}-F_{N1}=0,$$

(2.20) $$-\psi f_{sX}+f_{sY}+\theta f_{sZ}+\psi F_{N1}=0.$$

Using the above two equations and two unknowns, we can find:


(2.21) $$F_{sY}+\theta f_{sZ}+\psi^{2}f_{sY}-\phi \psi f_{sZ}=0,$$

(2.22) $$F_{N1}=f_{sX}+\psi f_{sY}-\phi f_{sZ},$$

(2.23) $$\begin{array}{r} \frac{w^{\prime }}{2}\mu (k_{X}\left(\delta_{X}+X-X_{0}\right)+\psi k_{Y}\left(\delta_{Y}+Y-Y_{0}\right)-\phi k_{Z}\left(\delta_{Z}+Z-Z_{0}\right))\\ +\left(H+L_{C}-h\right)\psi (k_{X}\left(\delta_{X}+X-X_{0}\right)+\psi k_{Y}\left(\delta_{Y}+Y-Y_{0}\right)-\phi k_{Z}\left(\delta_{Z}+Z-Z_{0}\right))\\ +k_{\theta }\left(\delta_{\theta }+\theta -\theta_{0}\right)+\psi k_{\phi }\left(\delta_{\phi }+\phi -\phi_{0}\right)-\phi \left(\delta_{\psi }+\psi -\psi_{0}\right)=0. \end{array}$$

Now, a quadratic equation can be formulated to determine the roots corresponding to the extraction depth *h*:


(2.24) $$\begin{array}{ccc} & ah^{2}+bh+c=0 & \end{array}$$

in which


$$\begin{array}{r} a≜k_{Y}\left(\delta_{Y}+2w-\frac{3w^{\prime }}{2}-Y_{0}\right), \end{array}$$

$$\begin{array}{r} b≜-\frac{w^{\prime }}{2}\mu k_{Y}\left(\delta_{Y}+2w-\frac{3w^{\prime }}{2}-Y_{0}\right)-k_{Y}\left(\delta_{Y}+2w-\frac{3w^{\prime }}{2}-Y_{0}\right)\left(H+L_{C}\right)\\ +k_{Y}\left(w-w^{\prime }\right)\left(H+L_{C}\right)-\left(w-w^{\prime }\right)\mu k_{X}\left(\delta_{X}-\frac{v^{\prime }}{2}-X_{0}\right)\frac{1}{2}+k_{\theta }\left(\delta_{\theta }-\theta_{0}\right), \end{array}$$

$$\begin{array}{r} c≜\frac{w^{\prime }}{2}\mu k_{Y}\left(w-w^{\prime }\right)\left(H+L_{C}\right)+\frac{w^{\prime }}{2}\mu \left(w-w^{\prime }\right)\mu k_{X}\left(\delta_{X}-\frac{v^{\prime }}{2}-X_{0}\right)\\ +k_{Y}\left(w-w^{\prime }\right){\left(H+L_{C}\right)}^{2}+k_{\theta }\left(w-w^{\prime }\right). \end{array}$$

The roots (extraction depth^1^ ) of the equation are


(2.25) $$h_{1}=\frac{-b+\sqrt{b^{2}-4ac}}{2a},\;h_{2}=\frac{-b-\sqrt{b^{2}-4ac}}{2a}.$$

The existence of *L*_*C*_ in equation (2.25) demonstrated its effect in the extraction depth and as a result extraction force.

#### 2.2.2. Case 2: two-sided contact

In this section, we conduct an analysis of the contact state wherein two lines of the peg come into contact with the inner surfaces of the hole (figure 4*b*). In this scenario, the rigidity inherent in both the peg and the hole results in a uniform distribution of the vertical force along the contact lines. Consequently, the vertical force and the friction force, which are initially distributed forces, are treated as concentrated forces acting at the centre of the contact lines.

Based on the relative position of the peg in relation to the hole, kinematic constraints can be delineated as follows. Kinematic constraints represent equations that govern the motion of solids, faces, edges or points and can be formulated utilizing predefined coordinate systems or custom coordinate systems.


(2.26) $$R_{1}-T_{p}^{h}r_{1}=R_{C},\;R_{2}-T_{p}^{h}r_{2}=R_{C}.$$

Let $R_{C}=$ [X, Y, Z]′, $R_{1}$ and $R_{2}$ denote the midpoints of two contact lines in the hole coordinates, and $r_{1}$ and $r_{2}$ represent the midpoints of two contact lines in the peg coordinates. In this scenario, it is evident that ψ=0, and ϕ is contingent on the extraction depth, denoted as *h*. The calculation for ϕ is as follows:


(2.27) $$v=h\sin\phi +v^{\prime }\cos\phi .$$

For small clearances and angles between pegs and holes, equation (2.27) can be simplified as $v=h\phi +v^{{,}^{\prime }}$and therefore


(2.28) $$\begin{array}{r} \phi =\frac{v-v^{\prime }}{h}. \end{array}$$

Using the same assumption, the coordinate transformation matrix is also calculated as follows:


(2.29) $$T_{p}^{h}=\left[\begin{array}{ccc} c\phi & 0 & s\phi \\ 0 & 1 & 0\\ -s\phi & 0 & c\phi \end{array}\right],\;T_{h}^{p}=\left[\begin{array}{ccc} c\phi & 0 & -s\phi \\ 0 & 1 & 0\\ s\phi & 0 & c\phi \end{array}\right].$$

Additionally, the peg exhibits an offset in the *Y*-direction from the hole wall, denoted as ε. Consequently, the following equations can be formulated:


$$\begin{array}{r} R_{1}=\left(\varepsilon +\frac{w^{\prime }}{2}\right)\hat{j},\;R_{2}=\left(\varepsilon +\frac{w^{\prime }}{2}\right)\hat{j}-v^{\prime }\hat{i}-h\hat{k}, \end{array}$$

(2.30) $$r_{1}=\frac{v^{\prime }}{2}\hat{i}-\left(H+L_{C}-h\right)\hat{k},\;r_{2}=-\frac{v^{\prime }}{2}\hat{i}-\left(H+L_{C}\right)\hat{k}.$$

Using equation (2.26), $R_{C}$ will result in


(2.31) $$R_{C}=\left[\begin{array}{c} s\phi \left(H+L_{C}-h\right)-\frac{c\phi v^{\prime }}{2}\\ \varepsilon +\frac{w^{\prime }}{2}\\ c\phi \left(H+L_{C}-h\right)+\frac{s\phi v^{\prime }}{2} \end{array}\right]=\left[\begin{array}{c} X\\ Y\\ Z \end{array}\right].$$

**Figure 8.**
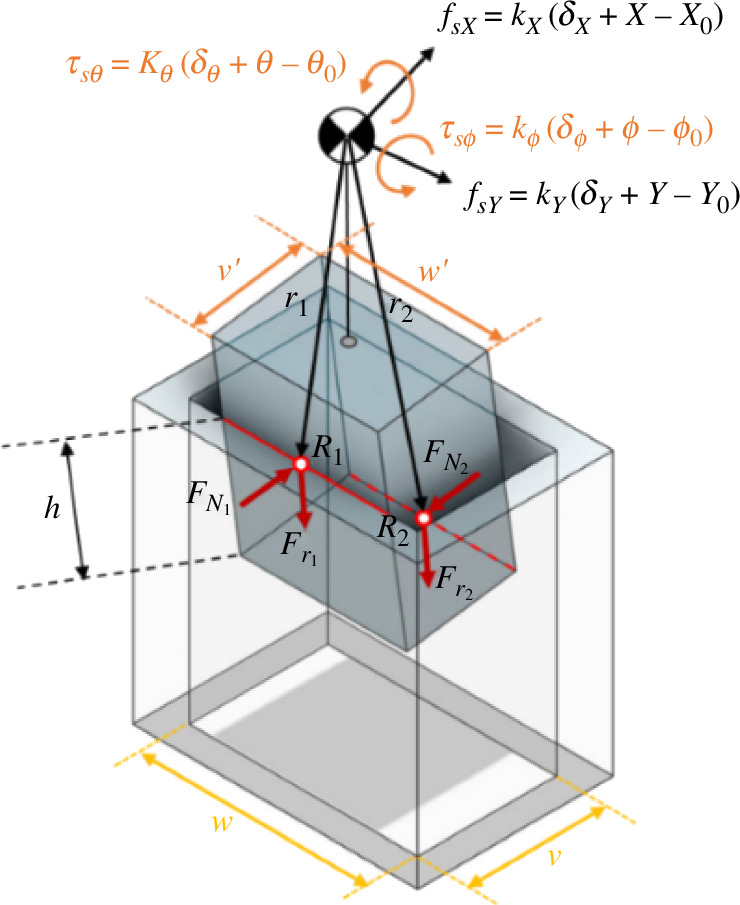
Geometry and forces during two-sided contact in the rectangular peg–hole.

A comprehensive force analysis can be conducted by considering the forces and torques arising from the peg–hole contact, alongside the forces and torques at the compliance centre attributed to spring length variations. The force $F_{z}$ can therefore be determined by other relevant variables (figure 8).

Considering $\sum F_{p}=0$ and $\sum M_{p}=0$ and doing the same calculations as for Case 1, the extraction depth^2^ *h* is calculated as^3^


(2.32) $$h_{1}=\frac{-b+\sqrt{b^{2}-4ac}}{2a},\;h_{2}=\frac{-b-\sqrt{b^{2}-4ac}}{2a}$$

in which


$$a≜k_{X}\left(\delta_{X}-v-X_{0}\right),$$

$$b≜k_{X}\left(v-v^{\prime }\right)\left(H+L_{C}\right)-k_{\phi }\left(\delta_{\phi }-\phi_{0}\right)-k_{\phi }\left(v-v^{\prime }\right)-k_{X}\left(H+L_{C}+\frac{\mu v^{\prime }}{2}\right)\left(\delta_{X}-v-X_{0}\right),$$

$$c≜-\left(H+L_{C}+\frac{\mu v^{\prime }}{2}\right)k_{X}\left(v-v^{\prime }\right)\left(H+L_{C}\right).$$

The existence of *L*_*C*_ in equation (2.32) demonstrated its effect in the extraction depth and as a result extraction force.

#### 2.2.3. Case 3: two-sided and one-plane contact

In this section, we scrutinize the contact scenario where two lines and one plane of the peg contact the inner surfaces of the hole (figure 4*c*). In the context of plane contact, the vertical force of the surface is oriented perpendicular to both planes, and the friction force direction exhibits two components within the contact plane, which are determined by the direction of movement. However, due to the small angles involved, the direction of the friction force can be approximated as vertical. Consequently, all forces are applied at the centre of the contact surface, considering the uniformity of the vertical force along the contact lines. Additionally, both the vertical force and the friction force, which are initially distributed forces, are treated as concentrated forces applied at the centre of the contact lines.

A kinematic constraint/kinematic loop can be defined according to the position of the peg in relation to the hole:


(2.33) $$R_{1}-T_{p}^{h}r_{1}=R_{C},\;R_{2}-T_{p}^{h}r_{2}=R_{C},$$

where


(2.34) $$r_{1}={\left[\begin{array}{c} 0\\ -\frac{w^{\prime }}{2}\\ -\left(H+L_{C}\right) \end{array}\right]}_{p},\;r_{2}={\left[\begin{array}{c} 0\\ \frac{w^{\prime }}{2}\\ -\left(H+L_{C}-h\right) \end{array}\right]}_{p},\;r_{3}={\left[\begin{array}{c} \frac{v^{\prime }}{2}\\ -\frac{w^{\prime }}{2}+\bar{y}\\ -\left(H+L_{C}-\bar{z}\right) \end{array}\right]}_{p}.$$

Here, y and z denote the centre of the third contact surface in relation to the bottom corner of the peg. The contact surface consistently takes the shape of a trapezoid with a side length of *h* and height of $w^{{,}^{\prime }}$. However, given the negligible rotation angle of the peg within the hole, the contact surface can be approximated as a rectangle with sides *h* and $w^{{,}^{\prime }}$:


(2.35) $$\bar{y}=\frac{w^{\prime }}{2},\;\bar{z}=\frac{h}{2},\;r_{3}={\left[\begin{array}{c} \frac{v^{\prime }}{2}\\ 0\\ -\left(H+L_{C}-\frac{h}{2}\right) \end{array}\right]}_{p}.$$

**Figure 9.**
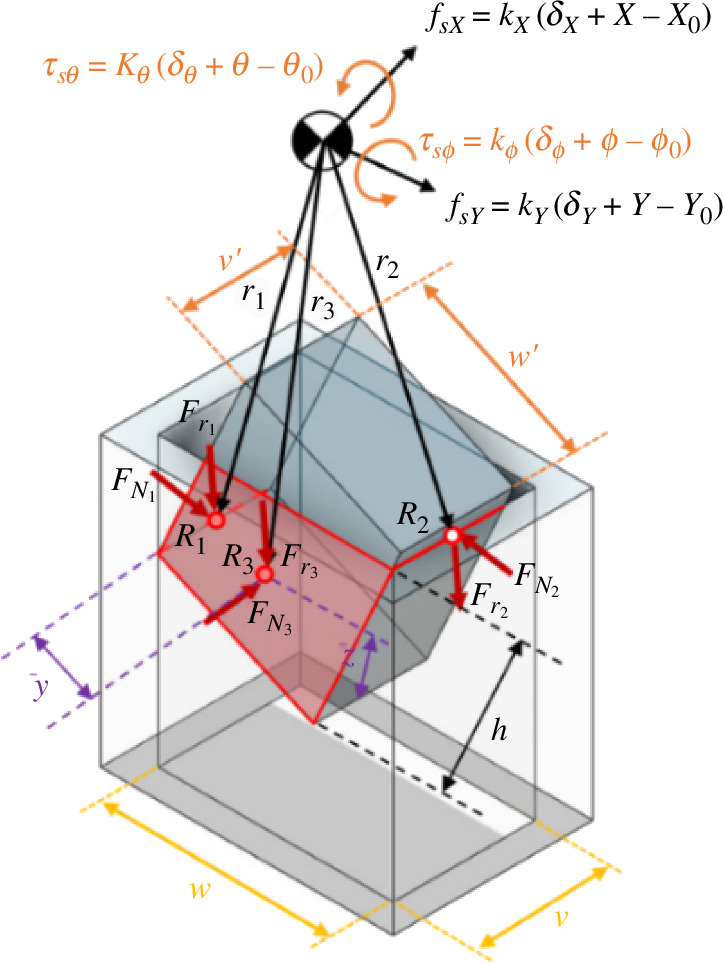
Geometry and forces during two-sided and one-plane contact in the rectangular peg–hole.


As illustrated in figure 9, the peg is constrained to rotate solely around adapter *X* to preserve the contact state. Consequently, ϕ and ψ are both zero, and θ can be expressed in terms of *h* as follows:

(2.36) $$h\theta =w-w^{\prime },\;\theta =\frac{w-w^{\prime }}{h}.$$

The coordinate transformation matrix and compliance centre position are calculated as follows:


(2.37) $$T_{p}^{h}=\left[\begin{array}{ccc} 1 & 0 & 0\\ 0 & c\theta & -s\theta \\ 0 & s\theta & c\theta \end{array}\right],\;T_{h}^{p}=\left[\begin{array}{ccc} 1 & 0 & 0\\ 0 & c\theta & s\theta \\ 0 & -s\theta & c\theta \end{array}\right],$$

(2.38) $$R_{C}=R_{2}-T_{p}^{h}r_{2}=\left[\begin{array}{c} -\frac{v^{\prime }}{2}\\ w-\frac{w^{\prime }}{2}-\theta \left(H+L_{C}-h\right)\\ -\theta \frac{w^{\prime }}{2}+\left(H+L_{C}-h\right) \end{array}\right]=\left[\begin{array}{c} X\\ Y\\ Z \end{array}\right].$$

A thorough force analysis can be undertaken by considering the forces and torques originating from the peg–hole contact, as well as the forces and torques at the compliance centre associated with variations in spring length. Consequently, the force $F_{z}$ can be expressed in terms of other pertinent variables.

Considering $\sum F_{p}=0$ and $\sum M_{p}=0$ and doing the same calculations as for Case 1, parameters of the extraction depth (*h*) would be calculated as^4^


$$a≜k_{Y}\left(\delta_{Y}+2w-\frac{3w^{\prime }}{2}-Y_{0}\right),$$

$$\begin{array}{r} b≜-\frac{w^{\prime }}{2}\mu k_{Y}\left(\delta_{Y}+2w-\frac{3w^{\prime }}{2}-Y_{0}\right)-k_{Y}\left(\delta_{Y}+2w-\frac{3w^{\prime }}{2}-Y_{0}\right)\left(H+L_{C}\right)\\ +k_{Y}\left(w-w^{\prime }\right)\left(H+L_{C}\right)-\left(w-w^{\prime }\right)\mu k_{X}\left(\delta_{X}-\frac{v^{\prime }}{2}-X_{0}\right)\frac{1}{2}+k_{\theta }\left(\delta_{\theta }-\theta_{0}\right), \end{array}$$

$$\begin{array}{r} c≜\frac{w^{\prime }}{2}\mu k_{Y}\left(w-w^{\prime }\right)\left(H+L_{C}\right)+\frac{w^{\prime }}{2}\mu \left(w-w^{\prime }\right)\mu k_{X}\left(\delta_{X}-\frac{v^{\prime }}{2}-X_{0}\right)\\ +k_{Y}\left(w-w^{\prime }\right){\left(H+L_{C}\right)}^{2}+k_{\theta }\left(w-w^{\prime }\right). \end{array}$$

Similarly, from the parameters it is obvious that the extraction depth and force are dependent on the location of $L_{C}.$

#### 2.2.4. Case 4: two-edge contact

In this section, we analyse the contact scenario in which two edges of the peg come into contact with the surfaces of the hole (figure 4*d*). Both the vertical force and the friction force initially span along the contact lines but are treated as concentrated forces positioned at the midpoint of the contact line. The maintenance of the contact state involves the peg moving both vertically and in the *Y* direction. In this scenario, the angles θ and ϕ are negligible, and for simplification, we consider them to be zero.

In this case, the coordinate transformation matrix is formulated as follows:


(2.39) $$T_{p}^{h}=\left[\begin{array}{ccc} c\psi & -s\psi & 0\\ s\psi & c\psi & 0\\ 0 & 0 & 1 \end{array}\right],\;T_{h}^{p}=\left[\begin{array}{ccc} c\psi & s\psi & 0\\ -s\psi & c\psi & 0\\ 0 & 0 & 1 \end{array}\right].$$

Additionally,


(2.40) $$w^{\prime }\sin\psi +v^{\prime }\cos\psi =v.$$

The ψ angle can be obtained in this manner.

A kinematic constraint or kinematic loop can be characterized based on the spatial arrangement of the peg relative to the hole:


(2.41) $$R_{1}-T_{p}^{h}r_{1}=R_{C},\;R_{2}-T_{p}^{h}r_{2}=R_{C},$$

where


(2.42) $$r_{1}={\left[\begin{array}{c} \frac{v^{\prime }}{2}\\ -\frac{w^{\prime }}{2}\\ -\left(H+L_{C}-\frac{h}{2}\right) \end{array}\right]}_{p},\;r_{2}={\left[\begin{array}{c} -\frac{v^{\prime }}{2}\\ \frac{w^{\prime }}{2}\\ -\left(H+L_{C}-\frac{h}{2}\right) \end{array}\right]}_{p}.$$

The forthcoming steps involve the calculation of the coordinate transformation matrix and the determination of the compliance centre position through the following procedures:


(2.43) $$R_{C}=R_{1}-T_{p}^{h}r_{1}=\left[\begin{array}{c} -\frac{v^{\prime }}{2}-\psi \frac{w^{\prime }}{2}\\ \varepsilon +\frac{w^{\prime }}{2}-\psi \frac{v^{\prime }}{2}\\ H+L_{C}-h \end{array}\right]=\left[\begin{array}{c} X\\ Y\\ Z \end{array}\right].$$

**Figure 10.**
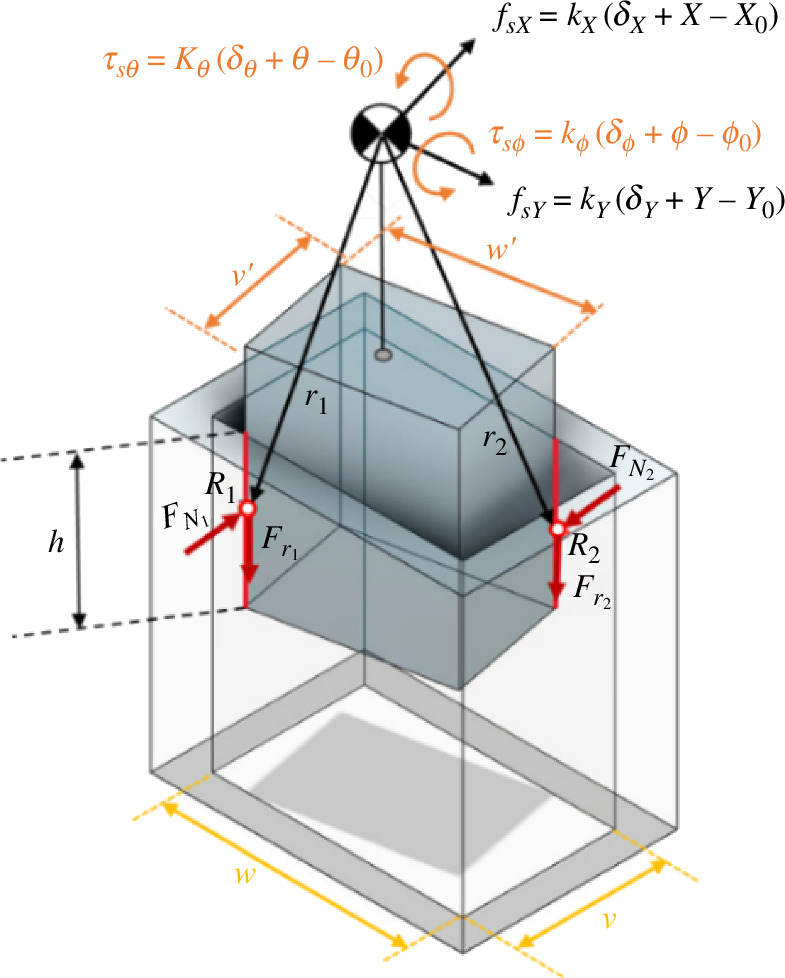
Geometry and forces during two-edge contact in a rectangular peg–hole.

Anticipating the ensuing steps, an exhaustive force analysis entails the consideration of forces and torques stemming from the interaction between the peg and the hole, along with those affecting the compliance centre due to variations in spring length. The outcome of this analysis will facilitate the determination of the force $F_{z}$ relative to other pertinent variables (figure 10).

Considering $\sum F_{p}=0$ and $\sum M_{p}=0$ the contact boundary condition is evidently dependent solely on the ψ angle.^5^ By formulating a cubic equation ($a\psi^{3}+b\psi^{2}+c\psi +d=0$) we can determine the corresponding roots of the angle ψ :


$$a≜-\frac{k_{Y}{v^{\prime }}^{2}}{2},$$

$$b≜-\frac{k_{X}v^{\prime }w^{\prime }}{2}+k_{Y}v^{\prime }\left(\delta_{Y}+\varepsilon +\frac{w^{\prime }}{2}-Y_{0}\right)+\frac{k_{Y}w^{\prime }v^{\prime }}{2},$$

$$c≜k_{X}v^{\prime }\left(\delta_{X}-\frac{v^{\prime }}{2}-X_{0}\right)+\frac{k_{X}{w^{\prime }}^{2}}{2}-k_{Y}w^{\prime }\left(\delta_{Y}+\varepsilon +\frac{w^{\prime }}{2}-Y_{0}\right)-2k_{\psi },$$

$$d≜-k_{X}w^{\prime }\left(\delta_{X}-\frac{v^{\prime }}{2}-X_{0}\right)-2k_{\psi }\left(\delta_{\psi }-\psi_{0}\right).$$

It is obvious that, in this particular case, the roots of the quadratic equation remain unaffected by the ACC’s location, indicating a unique scenario where the contact geometry dominates the extraction dynamics. This observation underscores the robustness and independence of the extraction process from the ACC position in this specific contact configuration. In other words, the position of the ACC becomes inconsequential to the extraction depth.

#### 2.2.5. Case 5: three-edge contact

In this section, we investigate the contact situation where three edges of the peg make contact with the surfaces of the hole (figure 4*e*). Initially, both the vertical force and the friction force extend along the contact lines but are simplified as concentrated forces located at the midpoint of the contact line. Preserving the contact state requires vertical and lateral movement of the peg. In this context, the angles θ and ϕ are deemed negligible, and for simplicity, we assume that their values are zero.

In this case, the coordinate transformation matrix is formulated as follows:


(2.44) $$T_{p}^{h}=\left[\begin{array}{ccc} c\psi & -s\psi & 0\\ s\psi & c\psi & 0\\ 0 & 0 & 1 \end{array}\right],\;T_{h}^{p}=\left[\begin{array}{ccc} c\psi & s\psi & 0\\ -s\psi & c\psi & 0\\ 0 & 0 & 1 \end{array}\right].$$

Additionally,


(2.45) $$w^{\prime }\sin\psi +v^{\prime }\cos\psi =v.$$

A kinematic constraint or kinematic loop can be characterized based on the spatial arrangement of the peg relative to the hole:


(2.46) $$R_{1}-T_{p}^{h}r_{1}=R_{C},\;R_{2}-T_{p}^{h}r_{2}=R_{C},\;R_{3}-T_{p}^{h}r_{3}=R_{C},$$

where


(2.47) $$r_{1}={\left[\begin{array}{c} \frac{v^{\prime }}{2}\\ -\frac{w^{\prime }}{2}\\ -\left(H+L_{C}-\frac{h}{2}\right) \end{array}\right]}_{p},\;r_{2}={\left[\begin{array}{c} -\frac{v^{\prime }}{2}\\ \frac{w^{\prime }}{2}\\ -\left(H+L_{C}-\frac{h}{2}\right) \end{array}\right]}_{p},\;r_{3}={\left[\begin{array}{c} -\frac{v^{\prime }}{2}\\ -\frac{w^{\prime }}{2}\\ -\left(H+L_{C}-\frac{h}{2}\right) \end{array}\right]}_{p}.$$

The upcoming stages encompass the computation of the coordinate transformation matrix and establishing the position of the compliance centre, achieved through the subsequent processes:


(2.48) $$R_{C}=R_{3}-T_{p}^{h}r_{3}=\left[\begin{array}{c} -\psi \frac{w^{\prime }}{2}-\frac{v^{\prime }}{2}\\ \frac{v^{\prime }}{2}\psi +\frac{w^{\prime }}{2}\\ H+L_{C}-h \end{array}\right]=\left[\begin{array}{c} X\\ Y\\ Z \end{array}\right].$$

**Figure 11.**
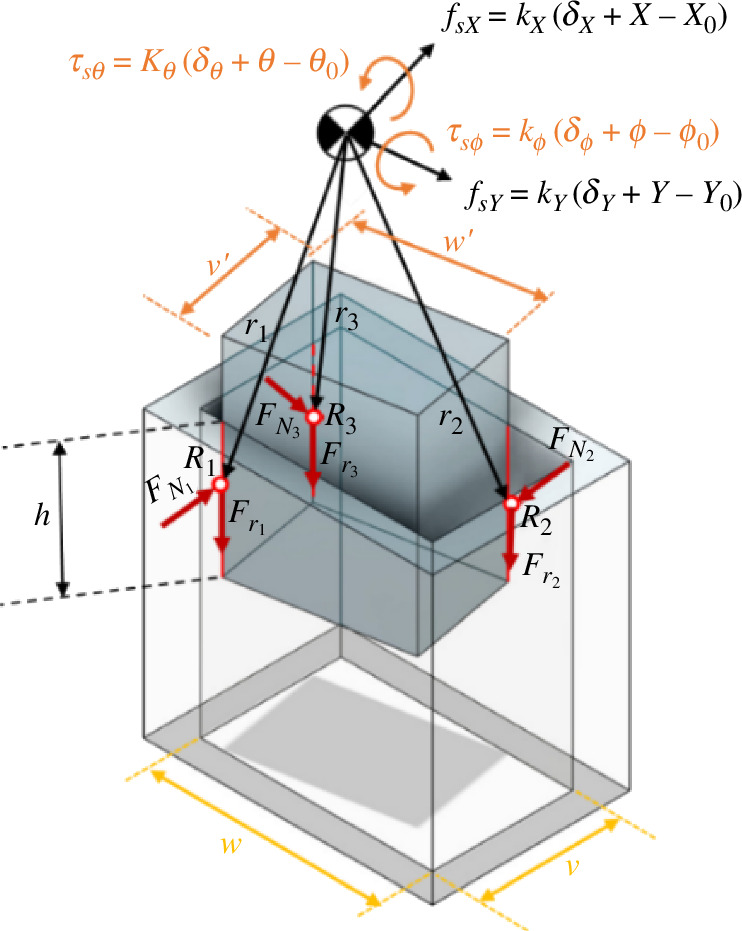
Geometry and forces during three-edge contact in the rectangular peg–hole.

Foreseeing the next phases, an in-depth examination of forces involves taking into account the forces and torques originating from the interaction between the peg and the hole, as well as those influencing the compliance centre due to changes in spring length. The results of this analysis will aid in establishing the force $F_{z}$ in relation to other relevant variables (figure 11).

The frictional force and the applied force are as follows:


(2.49) $$F_{N1}=-F_{N1}T_{h}^{p}\hat{i},\;F_{N2}=F_{N2}T_{h}^{p}\hat{i},\;F_{N3}=F_{N3}T_{h}^{p}\hat{j},\;F_{r1}=-\mu F_{N1}\hat{k},\;F_{r2}=-\mu F_{N2}\hat{k},\;F_{r3}=-\mu F_{N3}\hat{k}.$$

When the peg is being extracted from the hole, the contact line diminishes, resulting in a decrease in the friction force. As a result, the friction coefficient can be considered a function of *h* and is articulated as $\mu =\mu^{\prime }h$.

Considering $\sum F_{p}=0$ and $\sum M_{p}=0$ the contact boundary condition is evidently dependent solely on the ψ angle.^6^ By formulating $a\psi^{2}+b\psi +c=0$ as the quadratic equation, we have


$$a≜-k_{X}\frac{w^{\prime }}{2}+k_{Y}\frac{v^{\prime }}{2},$$

$$b≜k_{X}\left(\delta_{X}-X_{0}-\frac{v^{\prime }}{2}-\frac{w^{\prime }}{2}\right)+k_{Y}\left(\delta_{Y}-Y_{0}-\frac{v^{\prime }}{2}+\frac{w^{\prime }}{2}\right),$$

$$c≜k_{X}\left(\delta_{X}-X_{0}-\frac{v^{\prime }}{2}\right)-k_{Y}\left(\delta_{Y}-Y_{0}+\frac{w^{\prime }}{2}\right).$$

Same as Case 4, the roots of the quadratic equation remain unaffected by the ACC’s location.

## 3. Results and discussion

### 3.1. Region of extraction depth

The two roots in Cases 1, 2 and 3, $h_{1}$ and $h_{2}$, denote the depth of extraction (*h*). The two roots in Cases 4 and 5, $\psi_{1}$ and $\psi_{2}$, represent the rotation angles ψ around the *Z*-axis. Here, $h_{1}$ and $h_{2}$ represent the initial and final points of the two-contact region, respectively. If $h_{2}$ < $h_{0}$, the peg and hole are initially in the two-contact area at the commencement of the disassembly process. When $h_{0}$ < $h_{2}<h_{1}$, the peg–hole initially undergoes one contact, and there is at least one transition between the one-contact and two-contact states. If the equations have no solution, then no contact occurs during the extraction. When $h_{0}$ < $h_{2}=h_{1}$, the peg–hole exhibits a two-contact state at a specific depth. The dimensions and position of the two-contact region are influenced by various factors, including the geometric parameters of the hole–peg system, the compliance centre’s location, the initial position errors and the level of compliance.

### 3.2. Key factors

In the two-contact state, the reaction forces exerted on the peg increase the susceptibility to extraction failure, particularly at the entrance of the hole. Additionally, two-contact states contribute to an increase in the extraction force attributed to angular and lateral errors. This section delves into the identification of key parameters crucial for minimizing the two-contact region and elucidates their influence on its location. The parameters under scrutiny, namely the compliance centre position ($L_{C})$, initial lateral ($\delta_{X},\delta_{Y},\delta_{Z})$ and angular errors ($\delta_{\theta },\delta_{\Phi },\delta_{\psi })$ and stiffness ($K_{X},K_{Y}$, $K_{\theta },\;K_{\Phi }\;\text{and}\;K_{\psi }$), are subjected to analysis to elucidate their impacts on the two-contact region, with detailed discussions presented in subsequent sections.

#### 3.2.1. Location of the compliance centre

One crucial design parameter involves determining the optimal placement of the compliance centre along the central axis of the peg. When the manipulator secures a peg, initial positional discrepancies between the manipulator and the rectangular peg–hole system induce shifts and rotations in the peg. Assuming the rigidity of the rectangular peg–hole (figure 6), the blue figure illustrates the initial position of the peg before angular errors occur. However, initial lateral errors cause the peg to displace and rotate around the compliance centre (depicted in the red figure).

If the compliance centre is situated far from the peg tip, the peg rotates towards the contact side around the axis parallel to the contact line. For instance, if the intersection aligns with the length of the peg, rotation occurs around the *Y*-axis, potentially altering a single-contact state to a two-contact state. Conversely, when the compliance centre is positioned at the peg tip, rotation occurs around the parallel axis when gripping in the opposite direction to the line of contact, resulting in a single contact between the peg and the hole.

**Figure 12.**
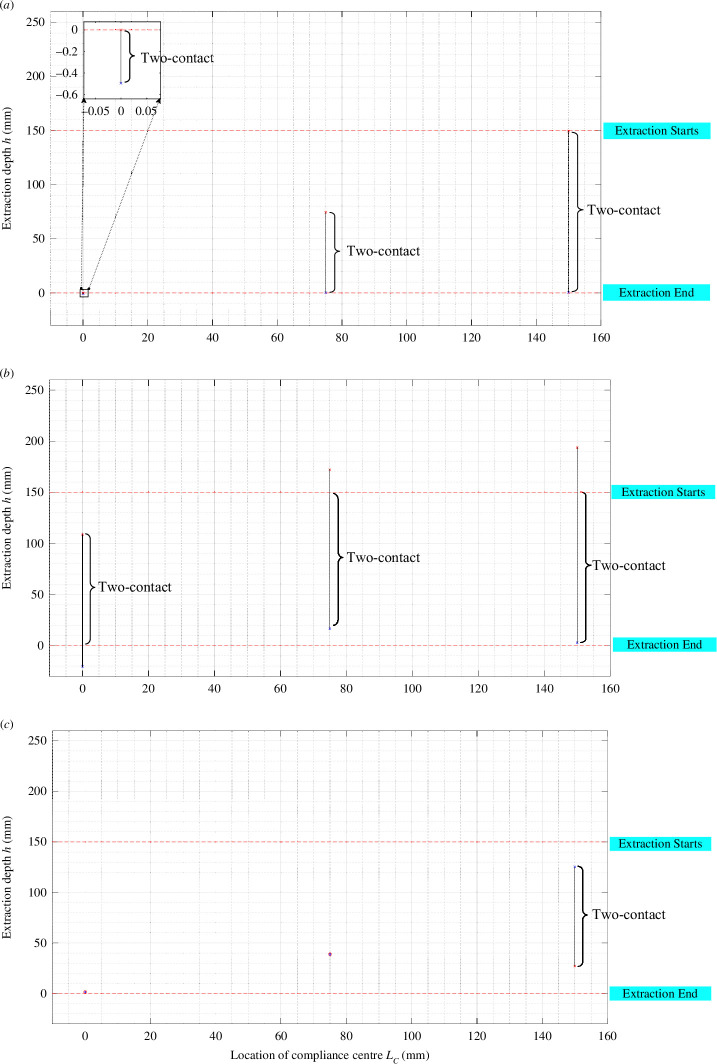
Dependence of the two-point contact region on the location of the compliance centre with $K_{X},K_{Y}=$ 3 N mm^−1^ and $K_{\theta },K_{\Phi },K_{\psi }=$ 30 N mm rad^−1^. (*a*) Two-point contact, (*b*) two-sided contact, (*c*) combined two-sided and one-plane contact.

These characteristics play a pivotal role in minimizing the two-point contact region during disassembly. As illustrated in figure 12, the boundary conditions of the two-contact region are presented based on the extraction depth equations ($h_{1}$ and $h_{2}$) for the initial three categories. It becomes evident that when the compliance centre is near the peg tip, the two-contact area is significantly reduced. Furthermore, as $L_{C}$ decreases, the two-point contact region shifts towards the hole mouth.

#### 3.2.2. Initial errors

**Figure 13.**
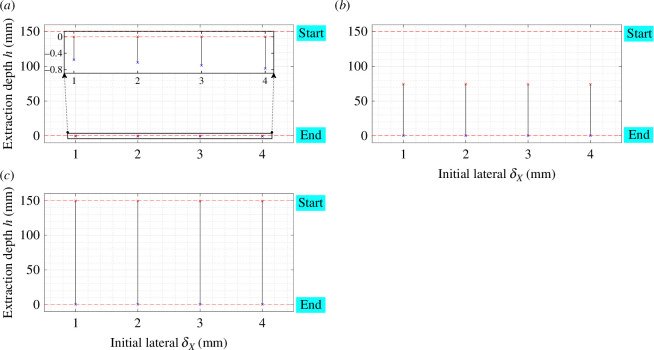
The effect of initial lateral error $\delta_{X}$ on the two-contact region in two-point contact with ${K_{X},K}_{Y}=$ 3 N mm^−1^, $K_{\theta },K_{\Phi },K_{\psi }=$ 30 N mm rad^−1^, and (*a*) $L_{C}=0\;,$ (*b*) $L_{C}=75\;\;mm,$ (*c*) $L_{C}=150\;\;mm.$ For the initial lateral (1–4), there was a decrease of 0.21% in the jamming contact region.

The two-contact region is susceptible to influence from initial position errors, encompassing lateral and angular deviations between the compliant manipulator and the peg–hole system. Notably, when the compliance centre is positioned far from the tip, substantial lateral errors ($\delta_{X}$ and $\delta_{Y}$) can induce significant rotation of the peg in the opposite direction to the axes of rotation. In instances where $\delta_{X}$ and $\delta_{Y}$ reach considerable magnitudes, the peg and hole persist in a two-contact region throughout the disassembly process. Conversely, if the compliance centre is situated at the tip, the peg rotates in alignment with the axes of rotation during disassembly. The transition from two-point contact to one-contact contact is observed as a consequence of these dynamics [13]. Figure 13 (electronic supplementary material, figures S1–S5) illustrates the impact of initial lateral errors on the two-contact region for diverse compliance centre locations. The two-contact region diminishes with increasing lateral errors when the compliance centre is in close proximity to the tip of the peg.

**Figure 14.**
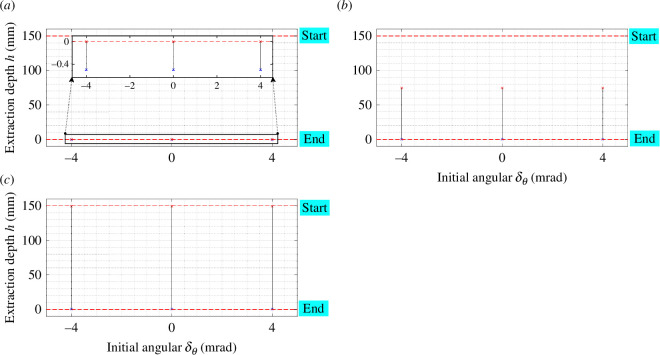
The effect of initial angular error $\delta_{\theta }$ on the two-contact region in the two-point contact with ${K_{X},K}_{Y}=$ 3 N mm^−1^, $K_{\theta },K_{\Phi },K_{\psi }=$ 30 N mm rad^−1^, and (*a*) $L_{C}=0\;,$ (*b*) $L_{C}=75\;mm,$ (*c*) $L_{C}=150\;mm.$

Angular errors ($\delta_{\theta },\;\delta_{\Phi }\;\text{and}\;\delta_{\psi }$) play a pivotal role in influencing the disassembly process in terms of both magnitude and direction. When the compliance centre is distant from the peg tip and the initial angular error opposes the peg’s rotation, as depicted in figure 14 (electronic supplementary material, figures S6–S13), a reduction in the two-contact region is evident. Conversely, if the initial angular error aligns with the rotation of the peg, the two-contact region expands. Similar effects of initial angular errors are observed when the compliance centre is located at the tip of the peg.

#### 3.2.3. Stiffness

**Figure 15.**
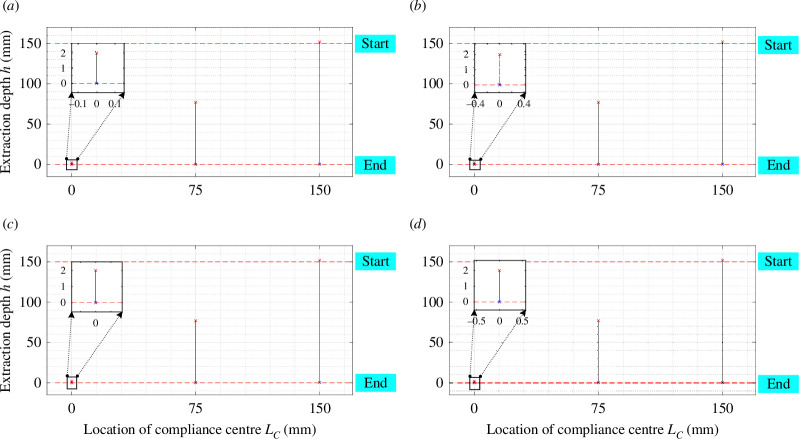
The effect of the two-contact region of the two-point contact on the structural parameters: (*a*) $K_{X}=$ 2 N mm^−1^; (*b*) $K_{X}=$ 3 N mm^−1^; (*c*) $K_{X}=$ 4 N mm^−1^; (*d*) $K_{X}=$ 6 N mm^−1^ with $K_{Y}=3\;{N\;mm}^{-1}$, $K_{\theta },K_{\Phi },K_{\psi }=$ 30 N mm rad^−1^, $\delta_{\theta },\delta_{\Phi },\delta_{\psi }=$ 0 rad.

**Figure 16.**
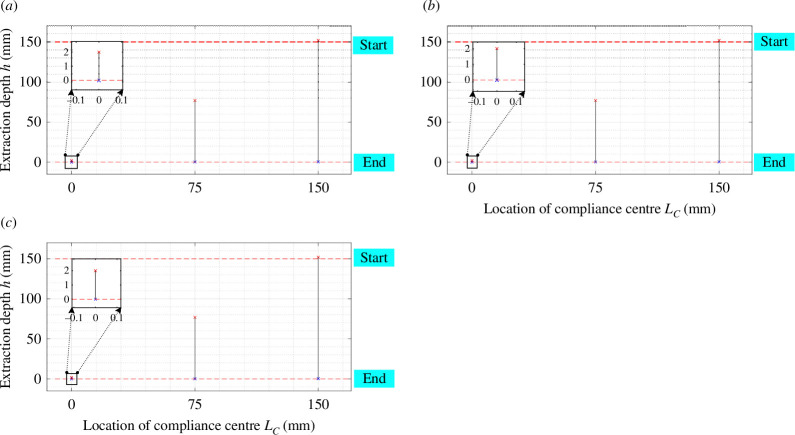
The effect of the two-contact region of the two-point contact on the structural parameters: (*a*) $K_{\theta }=$ 15 N mm rad^−1^; (*b*) $K_{\theta }=$ 30 N mm rad^−1^; (*c*) $K_{\theta }=$ 45 N mm rad^−1^ with $K_{X},\;K_{Y}=3\;{N\;mm}^{-1}$, $K_{\Phi },K_{\psi }=$ 30 N mm rad^−1^, $\delta_{\theta },\delta_{\Phi },\delta_{\psi }=$ 0 rad.

The success of a disassembly task is contingent upon the positioning of the compliance centre on the peg and the coupling stiffness elements between translational and rotational directions [6]. Compliant manipulators exhibit lateral and angular compliance determined by their rotational and lateral stiffness. Figure 15 (electronic supplementary material, figures S14–S18) and figure 16 (electronic supplementary material, figures S19–S26) show that as the lateral stiffness (${K_{X},K}_{Y}$ (N mm^−1^)) increases, the angular stiffness ($K_{\theta },K_{\Phi },K_{\psi }$ (N mm rad^−1^)) and the location of the compliance centre increase, and the two-contact region diminishes very slightly.

**Figure 17.**
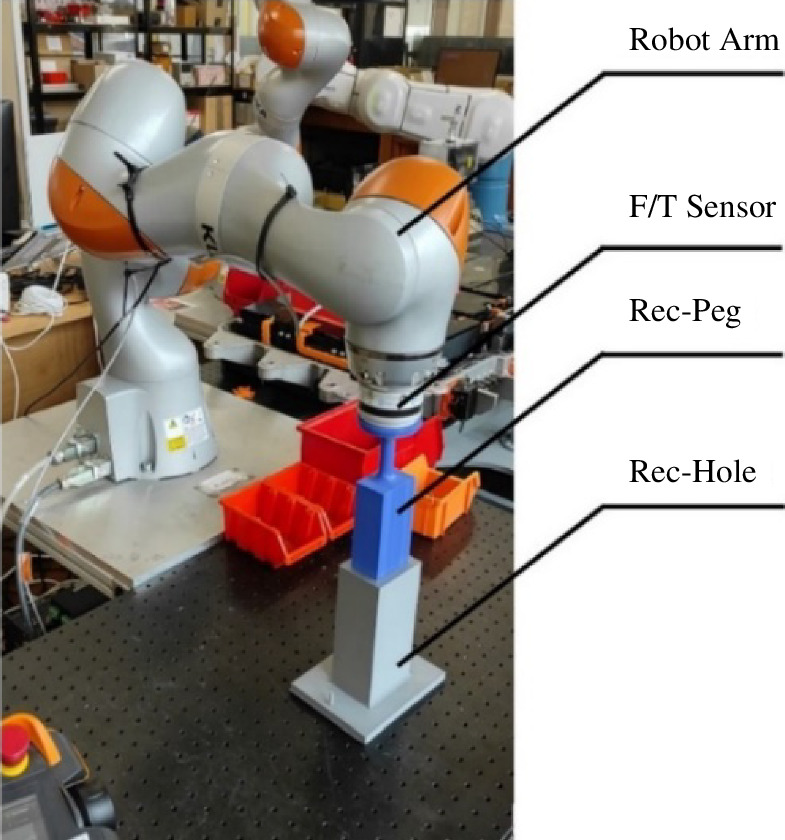
The experimental setup.

**Figure 18.**
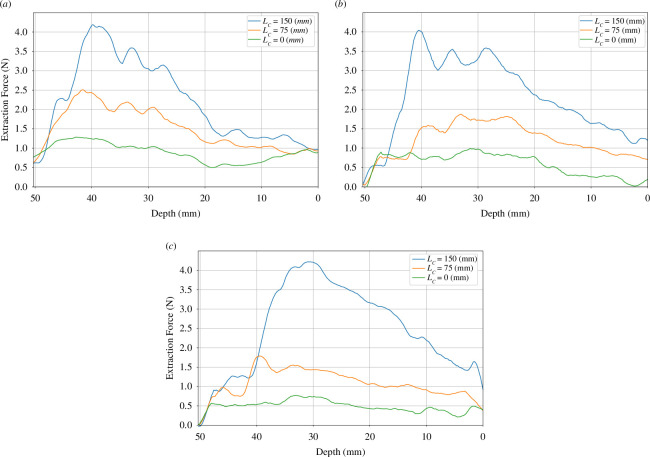
Extraction force curves based on compliance centre locations. (*a*) $w^{\prime }\times v^{\prime }$*=* 59.5 × 39.5 mm^2^, (*b*) $w^{\prime }\times v^{\prime }$ *=* 60 × 40 mm^2^, and (*c*) $w^{\prime }\times v^{\prime }$ *=* 60.5 × 40.5 mm^2^*.*

This phenomenon is attributed to heightened lateral stiffness, which results in reduced peg rotation for equivalent positional errors, thereby minimizing the two-point contact region. According to these figures, the stiffness has a limited impact on the two-contact region.

## 4. Experiment

In this section, an experiment is performed with three samples with different dimensions to verify the results of the above analysis. The geometric parameters of the rectangular peg–hole are given in table 2.

**Table 2.** Parameters for rectangular peg–hole.

category	parameter	value
friction characteristics	static coefficient of friction for PLA	$\mu_{s}=0.266$
	peg mass	*m* = 0.4 kg
geometrical parameters	peg dimensions	sample 1: 59.5 × 39.5 mm^2^
		sample 2: 60 × 40 mm^2^
		sample 3: 60.5 × 40.5 mm^2^
	hole dimensions	60.1 × 40.1 mm^2^
	peg length	*L* = 150 mm
initial position/angle	depth	$h_{0}=150\;mm$
	angle	$\theta_{0}=-0.9\;rad$

### 4.1. Experimental design

The experimental setup described in this section employed a six-degree-of-freedom KUKA LBR iiwa robot [24] in conjunction with a wrist-mounted force/torque sensor (figure 17). The video documenting the experiment is available at Figshare [25]. During the experiment, the force and moment data were recorded. The experimental setup involved placing a block with a hole on a table, with a peg affixed to the media flange of the robot arm. A noteworthy feature of this configuration is the deliberate exclusion of a gripper by attaching the workpiece to the robot’s end effector, as in the work by Zhang *et al*. [11].

Both the peg and the hole were fabricated from thermoplastic material. The experiment was conducted 18 times for each sample, with six repetitions for each compliance centre location ($L_{C}$ = 150 mm, $L_{C}$ = 75 mm, $L_{C}$ = 0 mm).

The tool centre point (TCP) was dynamically established through active compliance. In this context, the peg could rotate around the TCP. To experiment diverse compliance centre locations, various TCP configurations were implemented [24].

### 4.2. Experimental results

The experimental parameters utilized in this study can be found in table 2. To ensure consistency, a stiffness value of $K_{x},K_{y}=$ 3 N mm^−1^ was set along the extraction direction. Prior to plotting the extraction force measurements, a Savitzky‒Golay filter was employed to effectively eliminate any noise present in the data.

Figure 18 visually presents the outcomes obtained from investigating the influence of the location of the compliance centre on the extraction forces across various depths for three samples with different dimensions. As the extraction force increases, the area of the two contacts progressively expands. This observation highlights the significant disparity in contact states between theoretical predictions and practical implementations, depending on the position of the compliance centre.

Notably, the blue curve in figure 18 clearly indicates that the two-point contact region attains its maximum extent when the compliance centre is situated far from the tip of the rectangular peg. On the other hand, the orange and green curves demonstrate narrower and lower peaks as the distance $L_{C}$ decreases, thus corresponding to a diminished two-contact region.

## 5. Conclusion

This paper has conducted a thorough examination of the challenges associated with extracting a rectangular peg from a hole, focusing on geometric and quasistatic force analysis. Identifying 26 potential contact states in this scenario highlights complexities relevant to both the assembly and disassembly of rectangular peg–hole systems. The results of geometric and quasistatic force analysis provide valuable insights into the intricacies of jamming phenomena specific to rectangular peg–hole disassembly. Through systematic exploration involving factors such as compliance centre location, initial errors in lateral displacement or angular orientation, and manipulator stiffness, significant insights have been gained regarding their influence on two-contact states—emphasizing the crucial role of optimizing these aspects to enhance disassembly processes effectively.

Our findings demonstrated that positioning the ACC at the end of the peg results in the lowest extraction force compared with alternative ACC placements along the peg. Moreover, we have shown that allocating the ACC in this manner decreases or prevents the occurrence of jamming. To demonstrate the practical applications of our research, we utilized a 7 d.f. compliant manipulator, the KUKA LBR, in real-world settings. Over multiple experiments, we demonstrated that placing the ACC at the end of the peg reduced the average of maximum extraction force by 77.1% compared with the top of the peg position, and by 55.6% compared with the centre of the peg position, which suggests a more efficient method for engineers in robotic disassembly operations. These findings suggest that a robot with a reduced payload could be effectively utilized, provided it employs an appropriate ACC strategy. This means that in many disassembly cases, lighter robots with lower payload capacities can be sufficient instead of heavy, high-payload robots with a good ACC strategy.

In conclusion, this study not only enhances the understanding of disassembly processes but also underscores how the application of robotic systems in remanufacturing and other related industries can be revolutionized by integrating these findings. Our research focused on rectangular peg–hole systems which represent an elementary disassembly case. More complex disassembly cases, such as irregular geometries, can be investigated through combining this research into rectangular peg–hole systems and other elementary disassembly cases such as cylindrical peg–hole systems [11] and multi-peg–multi-hole systems [7,22]. Future work could expand upon adhesive force by incorporating such effects, potentially enhancing the model’s applicability to recycling and remanufacturing scenarios where such forces are more prominent. Additionally, research could investigate systems with changeable stiffness, exploring how dynamically adjusting stiffness parameters could improve the efficiency and adaptability of robotic systems during disassembly processes. These extensions may open new avenues for optimizing disassembly tasks in real-world applications.

## Data Availability

We have provided all the details of our paper, including the code, data and experiment video, at Figshare [26]. Supplementary material is available online [27].

## Footnotes

^1^
The period of the equation’s roots ($h_{1}$ and $h_{2}$) is discussed in §3.1.

^2^
The period of the equation’s roots ($h_{1}$ and $h_{2})$ is discussed in §3.1.

^3^
Detailed calculations in electronic supplementary material.

^4^
Detailed calculations in electronic supplementary material.

^5^
Detailed calculations in electronic supplementary material.

^6^
Detailed calculations in electronic supplementary material.

## References

[1] Goli F, Wang Y, Saadat M. 2022 Perspective of self-learning robotics for disassembly automation. In 2022 27th Int. Conf. on Automation and Computing (ICAC), *Bristol, UK, 1–3 September 2022*, pp. 1–6. (10.1109/ICAC55051.2022.9911085)

[2] Mule JY. 2012 Design for disassembly approaches on product development. Int. J. Sci. Eng. Res. **3**, 1–5.

[3] Ijomah WL, McMahon CA, Hammond GP, Newman ST. 2007 Development of robust design-for-remanufacturing guidelines to further the aims of sustainable development. Int. J. Prod. Res. **45**, 4513–4536. (10.1080/00207540701450138)

[4] Vongbunyong S, Chen WH. 2015 Disassembly automation. In Disassembly automation: automated systems with cognitive abilities, pp. 25–54. Cham, Switzerland: Springer. (10.1007/978-3-319-15183-0_3)

[5] Tang Y, Zhou M, Zussman E, Caudill R. 2002 Disassembly modeling, planning, and application. J. Manuf. Syst. **21**, 200–217. (10.1016/S0278-6125(02)80162-5)

[6] Kim BH, Yi BJ, Suh IH, Oh SR. Stiffness analysis for effective peg-in/out-hole tasks using multi-fingered robot hands. In Proc. 2000 IEEE/RSJ Int. Conf. on Intelligent Robots and Systems (IROS 2000), *Takamatsu, Japan, 31 October–5 November 2000*, pp. 1229–1236. (10.1109/IROS.2000.893187)

[7] Goli F, Zhang Y, Qu M, Zang Y, Saadat M, Pham DT, Wang Y. 2023 Jamming problems and the effects of compliance in dual peg-hole disassembly. Proc. R. Soc. A **480**, 20230364. (10.1098/rspa.2023.0364)

[8] Usubamatov R, Leong KW. 2011 Analyses of peg‐hole jamming in automatic assembly machines. Assem. Autom. **31**, 358–362. (10.1108/01445151111172943)

[9] Whitney DE. 1982 Quasi-static assembly of compliantly supported rigid parts. J. Dyn. Syst. Meas. Control **104**, 65–77. (10.1115/1.3149634)

[10] McNelly BP, Leary R, Brennan S, Reichard K. 2016 Characterizing successful robotic insertion and removal from a dry storage cask using peg-like jamming and wedging analysis. In ASME 2016 Pressure Vessels and Piping Conf., *Vancouver, Canada, 17–21 July 2016*. (10.1115/PVP2016-63634)

[11] Zhang Y, Lu H, Pham DT, Wang Y, Qu M, Lim J, Su S. 2019 Peg–hole disassembly using active compliance. R. Soc. Open Sci. **6**, 190476. (10.1098/rsos.190476) 31598244 PMC6731726

[12] Asada H, Kakumoto Y. 1988 The dynamic RCC hand for high-speed assembly. In Proc. 1988 IEEE Int. Conf. on Robotics and Automation, *Philadelphia, PA, USA, 24–29 April 1988*, pp. 120–125. (10.1109/ROBOT.1988.12035)

[13] Simunovic SN. 1979 An information approach to parts mating. Doctoral thesis, Massachusetts Institute of Technology, Cambridge, MA, USA.

[14] Nevins JL, Whitney DE. 1979 Assembly research. IFAC Proc. Vol. **12**, 195–214. (10.1016/S1474-6670(17)65359-X)

[15] Caine ME, Lozano-Perez T, Seering WP. 1989 Assembly strategies for chamferless parts. In Proc. 1989 Int. Conf. on Robotics and Automation, *Scottsdale, AZ, USA, 14–19 May 1989*, pp. 472–477. (10.1109/ROBOT.1989.100031)

[16] Sturges RH. 1988 A three-dimensional assembly task quantification with application to machine dexterity. Int. J. Rob. Res. **7**, 34–78. (10.1177/027836498800700403)

[17] Sturges RH, Laowattana S. 1996 Virtual wedging in three-dimensional peg insertion tasks. J. Mech. Des. **118**, 99–105. (10.1115/1.2826863)

[18] Sturges RH, Laowattana S. 1996 Design of an orthogonal compliance for polygonal peg insertion. J. Mech. Des. **118**, 106–114. (10.1115/1.2826840)

[19] Strip DR. 1988 Insertions using geometric analysis and hybrid force-position control: method and analysis. In Proc. 1988 IEEE Int. Conf. on Robotics and Automation, *Philadelphia, PA, USA, 24–29 April 1988*, pp. 1744–1751. (10.1109/ROBOT.1988.12318)

[20] Wang W, Loh RNK, Gu EY. 1998 Passive compliance versus active compliance in robot‐based automated assembly systems. Ind. Robot. Int. J. **25**, 48–57. (10.1108/01439919810196964)

[21] Lan F, Castellani M, Truong Pham D, Wang Y. 2023 On the correctness of using two-dimensional representations in the analysis of cylindrical peg-hole insertion and withdrawal. R. Soc. Open Sci. **10**, 221021. (10.1098/rsos.221021) 37650059 PMC10465190

[22] Goli F, Zhang Y, Wang Y, Saadat M. 2024 An analysis of dual peg-hole disassembly problems. In Advances in remanufacturing (eds M Fera, M Caterino, R Macchiaroli, DT Pham), pp. 483–500. Cham, Switzerland: Springer. (10.1007/978-3-031-52649-7_38)

[23] Trong DN, Betemps M, Jutard A. 1995 Analysis of dynamic assembly using passive compliance. In 1995 IEEE Int. Conf. on Robotics and Automation, *Nagoya, Japan, 21–27 May 1995*, pp. 1997–2002. (10.1109/ROBOT.1995.525556)

[24] 2016 System software kuka sunrise.os 1.11. Kuka Sunrise workbench 1.11. Operating and programming instructions for system integrators.

[25] Goli F, Aflakian A, Qu M, Zang Y, M S, Pham D, Wang Y. 2024 Rectangle peg-hole disassembly process with active compliance centre. See https://figshare.com/s/2f8db80aaa00a0eb60d2. 10.1098/rsos.240956 PMC11597411 39606586

[26] Figshare. 2024 Characterising the mechanics of rectangular peg-hole disassembly and the effect of the position of the active compliance centre on the extraction force. See https://figshare.com/s/2f8db80aaa00a0eb60d2. 10.1098/rsos.240956 PMC11597411 39606586

[27] Goli F, Aflakian A, Qu M, Zang Y, Saadat M, Pham D *et al*. 2024 Supplementary material from: Characterising the mechanics of rectangular peg-hole disassembly and the effect of the active compliance centre on the extraction force. Figshare. (10.6084/m9.figshare.c.7552186) PMC11597411 39606586

